# Management of Cardiac Tamponade: A Comperative Study between Echo-Guided Pericardiocentesis and Surgery—A Report of 100 Patients

**DOI:** 10.4061/2011/197838

**Published:** 2011-09-20

**Authors:** Hasan Ali Gumrukcuoglu, Dolunay Odabasi, Serkan Akdag, Hasan Ekim

**Affiliations:** ^1^Department of Cardiology, Yuzuncu Yil University, 65100 Van, Turkey; ^2^Department of Cardiovascular Surgery, Yuzuncu Yil University, 65100 Van, Turkey; ^3^Department of Cardiology, Van Yuksek Ihtisas Hospital, Van, Turkey

## Abstract

*Background*. Cardiac tamponade (CT) represents a life-threatening condition, and the optimal method of draining accumulated pericardial fluid remains controversial. We have reviewed 100 patients with CT at our institution over a five-year period and compared the results of echo-guided pericardiocentesis, primary surgical treatment, and surgical treatment following pericardiocentesis with regard to functional outcomes. *Methods*. The study group consisted of 100 patients with CT attending Yuzuncu Yil University from January 2005 to January 2010 who underwent one of the 3 treatment options (echo-guided pericardiocentesis, primary surgical treatment, and surgical treatment following pericardiocentesis). CT was defined by clinical and echocardiographic criteria. Data on medical history, characteristics of the pericardial fluid, treatment strategy, and follow-up data were collected. *Results*. Echo-guided pericardiocentesis was performed in 38 (38%) patients (Group A), primary surgical treatment was preformed in 36 (36%) patients (Group B), and surgical treatment following pericardiocentesis was performed in 26 (26%) patients (Group C). Idiopathic and malignant diseases were primary cause of tamponade (28% and 28%, resp.), followed by tuberculosis (14%). Total complication rates, 30-day mortality, and total mortality rates were highest in Group C. Recurrence of tamponade before 90 days was highest in Group A. *Conclusions*. According to our results, minimal invasive procedure echo-guided pericardiocentesis should be the first choice because of lower complication and mortality rates especially in idiopathic cases and in patients with hemodynamic instability. Surgical approach might be performed for traumatic cases, purulent, recurrent, or malign effusions with higher complication and mortality rates.

## 1. Introduction

Cardiac tamponade (CT) is a clinical syndrome characterized by hemodynamic abnormalities resulting from an increase in pericardial pressure due to accumulation of contents such as serous fluid, blood, and pus [1]. Idiopathic or viral pericarditis, iatrogenic injury (invasive procedure-related, post-CABG), trauma, malignancy, uremia, collagen vascular disease, tuberculosis, postmyocardial infarction, aortic dissection and bacterial infection may lead to CT [1]. In 1935, Beck described diagnostic triad for CT consisting of decreasing arterial pressure, increasing venous pressure, and quiet heart [2]. Increasing intrapericardial pressure leads to restriction of cardiac filling, reduction of stroke volume, and cardiac output [2, 3]. Clinical signs in patient with CT include hypotension, tachycardia, pulsus paradoxus, raised jugular venous pressure, muffled heart sounds, decreased electrocardiographic voltage, and enlarged cardiac silhouette on chest roentgenogram [3]. Echocardiography is considered the primary imaging modality for the evaluation of pericardial effusion because of its high sensitivity and specificity, lack of ionizing radiation, and low cost. Computerized tomography and magnetic resonance imaging are indicated when findings at echocardiography are inconclusive [4]. 

The treatment of CT is based on clinical presentation and may involve pericardial content removal by percutaneous pericardiocentesis, balloon pericardiotomy, or surgical drainage [5]. More recently, echocardiographic-guided pericardiocentesis has been demonstrated to be a safe and effective procedure that can be performed at the bedside [6]. However, if pericardial tissue is required for diagnosis or in the case of purulent pericarditis or recurrent effusions, surgical drainage may be the preferred treatment. Also, immediate surgical approach should be performed for traumatic hemopericardium [7]. We have reviewed all patients with cardiac tamponade in our institution over a five-year period to determine the causes of cardiac tamponade, clinical aspects, treatment modalities, and long-term followup.

## 2. Subjects and Methods

### 2.1. Patients

The study was approved by the local ethics committee in accordance with the Declaration of Helsinki. The medical records of all patients with cardiac tamponade of our hospital from January 2005 to January 2010 were reviewed. Cardiac tamponade was defined by clinical and echocardiographic criteria [8, 9]. Two-dimensional echocardiographic criteria of CT were early diastolic collapse of the right ventricle, late diastolic collapse of the right or left atrium, and plethora of the inferior vena cava with pericardial effusion [10]. Doppler echocardiographic criteria of CT were major increases of tricuspid E flow and major decreases of mitral E flow during inspiration [11]. In all cases, the location and distribution of the pericardial effusion leading to tamponade were confirmed by two-dimensional and doppler echocardiography (Vivid 3, GE Vingmed Ultrasound, Horten, Norway). Victims with CT due to penetrating trauma were excluded from study.

### 2.2. Procedures and Techniques

*Pericardiocentesis *was done by subxiphoid approach. Percutaneous drainage was initiated with an 8 cm, 18-gauge angiocatheter. When the pericardial sac was entered, the sheath was advanced and the needle withdrawn. A guide wire was then advanced through the angiocatheter, followed by a dilator and a 60 cm, 6 F pigtail catheter. Pericardial fluid was fully drained and submitted for culture and cytological analysis. The sheath position was readily confirmed by injecting a small amount of agitated saline. The effusion was initially drained completely, as assessed by repeated echocardiography. Subsequently, intermittent aspirations were performed as clinically indicated, usually every 4 to 6 hours, until the fluid return over a 24-hour period had decreased to less than 25 mL. Followup with two-dimensional echocardiographic assessment was satisfactory. The aspiration was not drained more than 1000 mL at initial time due to acute right ventricular dilatation and hypotensive shock [5, 11]. Major complications included any undesirable events occurring as a result of pericardiocentesis that required intervention such as need for emergency surgery, ventricular arrhythmia, and perforation of cardiac chamber, hemothorax, and pneumothorax. Minor complications were those that required no management, except appropriate monitoring and followup [11]. Recurrence was defined as reaccumulation of fluid requiring intervention and was further categorized as to whether it occurred within or beyond 90 days of the initial pericardiocentesis [12, 13].

#### 2.2.1. Surgical Drainage Procedures

*Subxiphoid surgical drainage technique *was done by subxiphoid approach. 

A 5–10 cm skin incision was made from the lower end of the sternum and extended caudally approximately 5 cm. The upper linea alba was divided at the midline and xiphoid sternum was resected. A portion of the anterior pericardium was excised. The pericardial effusion was aspirated and fluid sample was sent for histological examination. A 28 F caliber chest tube was inserted posteriorly to the pericardium and left until the following 3-4 days. 

*Pericardioperitoneal window technique *was done by local anesthesia. 

The diaphragm and inferior pericardium were incised to create a pericardioperitoneal window. The edges of windows were stitched with interlocking sutures using 4-0 polypropylene to prevent closure. Fluid and pericardium sample was sent for histological examination. A drainage tube was inserted into pericardial space and left until the following day. 

*Left anterior minthoracotomy technique *was done by general anesthesia. Patients were yielded at left lateral decubitis position. Chest is opened between 4th and 5th intercostal spaces. A 4-5 cm portion of the left pericardium was excised. Fluid and pericardium sample was sent for histological examination. A drainage tube was inserted into pleural space and left until the following 3-4 days.

### 2.3. Outcomes

Outcomes of interest included procedural success, major and minor complication rates, effusion recurrence rates, and survival. Pericardiocentesis was considered successful if the pericardial fluid was drained with relief of tamponade. Major complication is considered as an undesirable event occurring as a result of pericardiocentesis that required intervention such as need for emergency surgery, cardiogenic shock, ventricular arrhythmia, perforation of cardiac chamber (iatrogenic cardiac injury), hemothorax, and pneumothorax. Minor complication is considered an event that required no management except appropriate monitoring and followup (hypotension, low cardiac out-put, tachycardia, etc.).

### 2.4. Statistical Analyses

All statistical analyses were conducted using SPSS system version 10.0 (SPSS, Inc., Chicago, Ill., USA). Descriptive statistics are presented as means ± standard deviation (SD) or by frequency percentages. Univariate comparison of categoric variables was done using Z test analysis. Multivariate risk analysis identified factors responsible for increased risk of effusion recurrence. Statistical differences were considered significant if the probability was 0.05 or less.

## 3. Results

This study group consisted of 100 consecutive patients, 51 (51%) female and 49 (49%) male patients, ranging in age from 10 to 79 years with a mean age of 46.3 ± 21.9 years. In our series 18 patients were <18 years, 57 patients were 18–60 years, and 25 patients were >60 years old. There were differences between groups in age and sex (*P* ≤ 0.01). Initial complaints of patients were dyspnea 89%, palpitation 61%, pretibial edema 48%, tachycardia (>100 beats/min) 82%, QRS alternation 29%, low QRS voltage 81%. There were differences between groups in initial complaints of patients (*P* ≤ 0.01). Hemodynamic status of the patients were echocardiographic tamponade (3 cm fluid accumulation within pericardial sac confirmed by echocardiography) 48%, clinical tamponade 44%, and hemodynamic collapse 8%. There were differences between Group C to Group A and B in hemodynamic status of the patients (*P* ≤ 0.01). There were no differences between Group A and B in hemodynamic status of the patients. *Characteristics of patients were summarized in* Table 1. 

Effusion characteristics of patients were circumferential distribution 78%, and loculated distribution 22%. There were differences between Group B to Group A and C in distribution of effusion of the patients (*P* ≤ 0.01). There were no differences between Group A and C in distribution of effusion of the patients. Size of the effusions were large (echocardiographically >2 cm effusion) 76% and small (echocardiographically <2 cm effusion) 24%. There were differences between groups in size of effusion of the patients (*P* ≤ 0.01). Color of the effusions was; bloody 47%, serosanguineous 18%, and serous 35%. There were differences between groups in colors of the effusions of the patients (*P* ≤ 0.01). *Effusion characteristics of patients were summarized in* Table 2. 

Etiology of all patients was malignancy 28% (13 patients had lung malignancy, 3 patients breast, 4 patients thyroid papillary malignancy, 2 patients esophagus, 4 patients mesothelioma, and 2 patients had renal cell malignancy) postoperative 2%, cardiac perforation from invasive procedure 6%, infection 4%, connective tissue disease 2%, ischemic heart disease related 2%, idiopathic 28%, hypothyroidism 6%, acute rheumatismal fever 4%, tuberculosis 14%, and warfarin overdose 4%. Idiopathic (28%), malign (28%) and tuberculous (14%) pericardial effusions were more frequently seen. There were differences between groups in etiology of pericardial tamponade (*P* ≤ 0.01). Etiology of pericardial tamponade of 3 study groups were summarized in Table 3.

There were differences between Group B to Group A and C in major complications (*P* ≤ 0.01). There were no differences between Group A and C in major complications. There were differences between Group B to Group A and C in minor complications (*P* ≤ 0.01). There were no differences between Group A and C in minor complications. 

When the recurrences reviewed, there were 16 (16%) recurrences seen before 90 day. 10 recurrent cases in Group A (26%), 3 cases in Group B (8%), and 3 cases in Group C (11%) were seen. There were differences between groups in recurrences before 90 days (*P* ≤ 0.01). When the recurrences were reviewed according to etiologic causes; 6 of the patients were from tuberculosis (16%), 3 from malignancy (8%), and 1 patient diagnosed idiopathic (2%) in Group A. No patient was recurred from tuberculosis (0%) and malignancy (0%), 3 patients diagnosed idiopathic (8%) in Group B. Similarly, no patient was recurred from tuberculosis (0%), and malignancy (0%) 3 patients recurred with the diagnosis of idiopathic (11%) in Group C. When the 30-day mortality rates were reviewed, there were 5 (5%) patients seen. There were differences between groups in the 30-day mortality rates (*P* ≤ 0.01). Group A: 5% (2/38), Group B: 3% (1/36), and Group C: 7% (2/26). Exitus was seen in 12 patients. There were differences between groups in exitus rates (*P* ≤ 0.01). Group A: 13% (5/38) patients, Group B: 8% (3/36) patients, and Group C: 15% (4/26) patients. Complete followup was achieved in 93% (93) patients. The mean followup time for the population was 3.5 ± 0.4 years. *Complications, recurrences, and followup of patients were summarized in* Table 4. 

## 4. Discussion

Cardiac tamponade is a treatable cause of cardiogenic shock that can be rapidly fatal if unrecognized [13]. Patients with impending or early tamponade are usually anxious and may complain of dispnea and chest pain [13]. In our study, the predominant symptom of patients was dyspnea (89%) and the main finding was tachycardia (82%). These findings were consistent with other series [6, 14]. Cardiac tamponade have long been associated with low voltage of the 12-lead electrocardiogram (ECG), and the diagnostic accuracy, sensitivity, and specificity of this ECG finding have previously been reported [15]. Different mechanisms have been proposed to explain low QRS voltage associated with pericardial effusion and CT. These mechanisms include mechanic-electrical alterations of the myocardium *which is generally seen in chronic heart failure*, distance of the heart from body surface electrodes and reduction of cardiac size and volume [16]. Bruch and coworkers reviewed 43 patients with significant pericardial effusion, finding 61% of cardiac tamponade. Main ECG finding of this report was that low QRS voltage was present in the majority of subjects with CT [17]. 81% of patients had low QRS voltage in our study population. Hemodynamic statuses of our patients were echocardiographic tamponade 48%, clinical tamponade 44%, and hemodynamic collapse 8%. In a Mayo clinic series that included 1127 patient echocardiographic tamponade rate was 49.8%, clinical tamponade rate was 44.2%, and hemodynamic collapse rate was 9.8% [6]. Allen and colleagues reported on a series of 117 patients with CT; etiologic causes were 64% malignancy (most often lung and breast), and benign disease 36% (most often idiopathic and uremic) [18]. In a Mayo clinic series etiologic causes of CT were malignancy (33%) and cardiac perforation from invasive procedure (10.3%) [6]. In our study, idiopathic (28%), malignant (28%), and tuberculous (14%) pericardial effusions were more frequently seen. The incidence of metastatic disease to the myocardium and/or pericardium ranges from less than 1 percent up to 18 percent of all cancer [19]. Lung and breast cancer which are the most common malignancies comprise nearly one-half of all metastatic lesions to the heart. Melanoma, leukemia, and lymphoma which occur less commonly, involve the pericardium in approximately 50 percent of patients [20–22]. In our series, most common malignancy was lung cancer. Tuberculosis is believed to be one of the main causes of pericarditis in developing countries [23]. Tuberculosis is diagnosed in 14% of patients in our series (Table 5). When CT results from hemorrhage into the pericardium, there can be rapid circulatory collapse because not only does intrapericardial pressure rapidly rise but intravascular volume falls, preventing a compensatory increase in venous pressure [5]. Therefore, during any invasive cardiologic intervention, operating room should be prepared due to possible risk of cardiac rupture [24]. In our series, cardiac tamponade and shock developed in 6 patients (6%) during invasive cardiologic procedure. They were operated on successfully without delay. Critical patients who presented with cardiac tamponade and pericardial effusion must be evacuated quickly. But which method to use for treatment is a controversial issue today. First implemented in 1841 pericardiocentesis with a needle, fixing the symptoms of patients but the permanent treatment cannot be provided [25]. This method in patients with tamponades is more applicable in intensive care and a less invasive method. However, mortality, complication, and recurrence rates are high. In a study by Kopecky and his colleagues with 42 patients there were no mortality, however, complication rate was 2.4% and recurrence rate was 24% [26]. In another study of Celermajer and his colleagues with 36 patients, mortality, complication, and recurrence rates were 3%, 5.6%, 19.4%, respectively [27]. Successful pericardiocentesis with a needle of tamponade in the study of Markiewicz 83% of patients had recurred [28]. Echo-guided pericardiocentesis technique was first reported as safe and effective by Mayo Clinic Group in pilot studies in the early 1980s [6]. In Mayo clinic follow-up series, echo-guided pericardiocentesis was confirmed to be safe and effective for treatment of clinically significant pericardial effusions. The procedural success rate was 97%. The overall total complication rate of 4.7% [6]. Our results are in accordance with the above-mentioned data (success rate 97%, total complication rate 10%). Echo-guided pericardiocentesis is well tolerated by patients, and can be performed quickly even in unstable patients. Symptoms associated with the pericardial effusion are relieved rapidly [6]. Pericardial catheters for extended drainage may remain in place without compromising patient mobility. The necessary equipment is widely available and portable, and the technique is adaptable to a broad spectrum of circumstances. Excellent results of echo-guided pericardiocentesis associated with emergency pericardiocentesis performed in critically unstable patients have been published [6, 28, 29]. For all these reasons, echo-guided pericardiocentesis appears to be a more practical and useful procedure than other reported percutaneous techniques [30]. Drainage of pericardial effusion by the subxiphoid region which was firstly described by Larrey in 1829 is the preferred method by many surgeons [31]. Treatment of patients with cardiac tamponades by subxifoid pericardial window method is simple and reliable [12, 14, 18, 32]. Even though the method has higher cost of implementation and is more invasive than percutaneous catheter drainage, it has more advantages to other techniques such as implementation chance under local anesthesia, visibility of tamponade directly from the pericardial cavity, and the opportunity of taking pericardial biopsy to clarify the etiology. Mortality, complications, and recurrence rates of patients with subxiphoid pericardial window are low [12, 14, 18, 32]. Surgical drainage procedures (partial or complete pericardiectomy, pericardial window) were popular in earlier years because of their association with low effusion recurrence rates [33]. However, the need for a general anesthesia in many of these procedures, with the attendant perioperative morbidity and mortality is undesirable. In a study of patients who underwent surgery for treatment of malignant effusion, a 30-day mortality rate of 19.4% was reported [33]. In another series involving 41 patients who underwent subxiphoid pericardiostomy, the 30-day mortality rate was 19.5% [34]. In our series 30 day mortality rates were as follows Group A: 5% (2/38), Group B: 3% (1/36), and Group C: 7% (2/26). Simple pericardiocentesis, without extended catheter drainage, has been associated with recurrence rates of up to 55% [6, 28]. In our series recurrences rates before 90 day rates were highest in Group A. When the recurrences were reviewed according to etiologic causes, we suggested that surgery should be performed rather than pericardiocentesis in the diagnosis of tuberculosis and malignancy. When the 30 day mortality rates were reviewed, we interpreted that surgery following pericardiocentesis has highest 30-day mortality rates. When the complications wer reviewed, we demonstrated that surgery has lower complication rates than other techniques by regarding the diagnosis. 12 patients died during followup, of whom 1 from coagulopathy, 2 from ischemic heart disease and cardiac failure, 1 from tuberculosis, and 8 from malignancy (Table 6).

## 5. Conclusions

The most common causes of CT were idiopathic and cancer. Echo-guided pericardiocentesis is safe, and the rate of complications has remained stable despite the fact that a greater number of procedures have been performed on an emergency basis. Surgery might be preferred in purulent, recurrent, and/or malign effusions and if pericardial biopsy is required for diagnosis. Also immediate surgical approach should be performed for traumatic hemopericardium, otherwise, less invasive procedure echo-guided pericardiocentesis might be the first choice especially in idiopathic cases and in patients with hemodynamic instability.

## Figures and Tables

**Table 1:** Characteristics of patients.

Group	Group A (*n* : 38)		Group B (*n* : 36)		Group C (*n* : 26)		Mean
Sex	Male (*n* : 17)	Female (*n* : 21)	Male (*n* : 16)	Female (*n* : 20)	Male (*n* : 16)	Female (*n* : 10)	*n* : 100
Age (year)	41.3 ± 26.5	35.8 ± 21.6	43.3 ± 21.5	47.9 ± 22.8	54.3 ± 32.3	55.7 ± 31.9	46.3 ± 21.9
Patients <18 years	3	4	2	3	4	2	18
Patients 18–60 years	9	11	9	13	8	7	57
Patients >60 years	5	6	5	4	4	1	25

Initial complaint of patients							

Dispnoea	16	17	14	18	15	9	89
Palpitation	4	11	12	14	14	6	61
Pretibial edema	3	7	12	14	6	6	48
Tachycardia (100 beats/min)	14	18	14	16	12	8	82
QRS alternation	7	6	4	5	4	3	29
Low QRS voltage	14	18	12	16	13	8	81

Hemodynamic status							

Echocardiographic tamponade	8	10	6	11	8	5	48
Clinical tamponade	9	8	8	8	7	4	44
Hemodynamic collapse	1	2	2	1	1	1	8

**Table 2:** Effusion characteristics of patients.

Group	Group A (*n* : 38)		Group B (*n* : 36)		Group C (*n* : 26)		Mean
Sex	Male (*n* : 18)	Female (*n* : 20)	Male (*n* : 16)	Female (*n* : 20)	Male (*n* : 16)	Female (*n* : 10)	*n* : 100

Distribution of effusion							

Circumferential	14	15	13	16	13	7	78
Loculated	4	5	3	4	3	3	22

Size of effusion							

Large (eco >2 cm) effusion	13	14	12	15	14	8	76
Small (eco <2 cm) effusion	5	6	4	5	2	2	24

Color of effusions							

Bloody	9	10	10	6	7	5	47
Serosanguineous	2	4	2	5	2	3	18
Serous	6	7	6	7	4	5	35

eco: echocardiography.

**Table 3:** Etiology of cardiac tamponade.

Group	Group A (*n* : 38)			Group B (*n* : 36)			Group C (*n* : 26)			Mean
Age groups	a	b	C	a	b	C	a	b	c	*n* : 100
Malignancy	0	0	3	1	9	5	0	6	4	28
Postoperative	0	0	0	0	1	0	0	1	0	2
Cardiac perforation from invasive procedure	0	0	0	2	4	0	0	0	0	6
Infection	0	0	0	1	2	1	0	0	0	4
Connective tissue disease	0	0	0	1	1	0	0	0	0	2
Ischemic heart disease related	0	0	0	0	1	1	0	0	0	2
Idiopathic	2	10	5	1	6	1	2	6	3	28
Hypotroidiea	3	3	0	2	2	0	0	0	0	6
Acute rheumatismal fewer	1	1	0	1	0	0	1	0	0	4
Tuberculosis	1	5	1	1	4	1	2	0	4	14
Warfarin overdose	0	2	1	0	3	0	0	1	0	4

(a) patients <18 years; (b) patients 18–60 years; (c) patients >60 years.

**Table 4:** Complications, recurrences, and followup of patients.

Group	Group A (*n* : 38)		Group B (*n* : 36)		Group C (*n* : 26)		Mean
Sex	Male (*n* : 18)	Female (*n* : 20)	Male (*n* : 16)	Female (*n* : 20)	Male (*n* : 16)	Female (*n* : 10)	*n* : 100

Total complication							
Major	1	1	0	1	1	1	5
Minor	1	1	1	0	2	3	8
Recurrence ≤90 day	6	4	1	2	2	1	16
30 day mortality rate	1	1	0	1	2	o	7
Exitus	2	3	1	2	3	1	12
Complete followup	17	19	16	18	15	9	93

Mean follow-up (year)	3.2 ± 0.5		3.6 ± 0.4		3.5 ± 0.5		3.5 ± 0.4

**Table 5:** Etiology of cardiac tamponade according to groups.

Group	M	P	C	I	Con	Isc	Id	H	A	T	W
A (*n* : 38)	3 (7%)	0	0	0	0	0	17 (44%)	6 (15%)	2 (7%)	6 (15%)	3 (7%)
B (*n* : 36)	15 (41%)	1 (2%)	6 (16%)	4 (11%)	2 (5%)	2 (5%)	3 (8%)	0	1 (2%)	1 (2%)	1 (2%)
C (*n* : 26)	10 (38%)	1 (3%)	0	0	0	0	8 (30%)	0	1 (3%)	6 (23%)	0
*P*	≤0.01	≤0.01	≤0.01	≤0.01	≤0.01	≤0.01	≤0.01	≤0.01	≤0.01	≤0.01	≤0.01

(M: malignancy, P: postoperative, C: cardiac perforation from invasive procedure, I: infection, Con: connectice tissue diseas, Isc: ischemic heart disease related, Id: idiopathic, H: hypotroidiea, A: acute rheomatismal fewer, T: tuberculosis, W: warfarin overdose).

**Table 6:** Complications, recurrences, and followup periods of patients according to groups.

	Group A (*n* : 38)	Group B (*n* : 36)	Group C (*n* : 26)	*P*
Complication	4 (10%)	2 (5%)	4 (15%)	≤0.01
Major	2 (5%)	1 (2.5%)	2 (7%)	≤0.01
Minor	2 (5%)	1 (2.5%)	2 (7%)	≤0.01
Recurrences and etiology	10 (26%)	3 (8%)	3 (11%)	≤0.01
Tuberculosis	6 (16%)	0	0	≤0.01
Malignancy	3 (8%)	0	0	≤0.01
İdiopathic	1 (2%)	3 (8%)	3 (11%)	≤0.01
30-day mortality rate	2 (5%)	1 (3%)	2 (8%)	≤0.01
Exitus	5 (13%)	3 (8%)	4 (15%)	≤0.01
Coagulopathy	1 (2.6%)	0	1 (4%)	≤0.01
Ischemic heart disease and cardiac failure	1 (2.6%)	0	0	≤0.01
Tuberculosis	1 (2.6%)	0	0	≤0.01
Malignancy	2 (5% )	3 (8%)	3 (11%)	≤0.01
Complete followup	36 (94%)	34 (94%)	24 (92%)	NS
